# Advanced deep learning-based brain tumor classification using a novel customized CNN and optimized residual network

**DOI:** 10.1371/journal.pone.0334430

**Published:** 2025-10-10

**Authors:** Mehwish Rasheed, Sajid Iqbal, Arfan Jaffar, Sheeraz Akram

**Affiliations:** 1 Faculty of Computer Science and Information Technology, The Superior University, Lahore, Pakistan; 2 Department of Computer Science and IT, The University of Lahore, Lahore, Pakistan; 3 Information Systems Department, College of Computer and Information Sciences, Imam Mohammad Ibn Saud Islamic University (IMSIU), Riyadh, Saudi Arabia; Prince Mohammad Bin Fahd University, SAUDI ARABIA

## Abstract

The uncontrollable and rapid growth of brain cells can lead to brain tumors. If left untreated, this condition may result in severe health consequences, including death. Accurate detection and classification are the essential steps toward understanding their mechanisms and ensuring effective treatment. Both tasks are challenging, with brain tumor detection being more complex due to variations in tumor size, structure, and location. Many scholars have employed machine learning and deep learning methods for brain tumor detection. Deep learning (DL) methods provide robust solutions for the detection and classification of brain tumors. Large volumes of healthcare imaging data can be analyzed using these techniques to identify and characterize tumors with high accuracy, often surpassing human performance. In this study, we propose two deep learning models, a novel customized Convolutional Neural Network (CNN) and an optimized ResNet101, to classify brain tumor images into four categories: gliomas, pituitary tumors, meningiomas, and no tumor. We used an MRI image dataset from Kaggle, consisting of 3,264 images. We performed five-fold cross-validation on the training and validation set, and a separate test set was used for final evaluation. The average training accuracy across the five-fold was 99.03±0.01% for the novel customized CNN and 99.87±0.03% for optimized ResNet101, and the average validation accuracy was 96.31±0.01% and 97.23±0.03%, respectively. After the cross-validation, the best-performing fold was then selected and evaluated on the test set, achieving training accuracies of 99.05%, 99.91% and testing accuracies of 97.72%, 98.73%, respectively. The optimized ResNet model achieved the highest performance among the two proposed models. Overall, these findings demonstrate the potential of deep learning models in supporting clinical decision-making for brain tumor classification, which may improve survival rates and human health outcomes.

## Introduction

Brain tumors are among the most life-threatening diseases, caused by the uncontrolled growth of abnormal cells within the cranial cavity. There are various types of brain tumors, such as gliomas, pituitary tumors, and meningiomas [1,2]. Gliomas are highly invasive brain tumors that arise from glial stem or progenitor cells and undergo rapid proliferation due to genetic and molecular alterations in undifferentiated neural tissue [3], whereas meningiomas originate in the meninges, the protective membranes surrounding the brain and spinal cord, and pituitary tumors are located centrally at the base of the brain [2]. These three tumor types differ in their location, size, and severity [4]. Therefore, it is imperative to observe the features of these tumor types to determine their pathological nature as benign or malignant. Approximately 80% of tumors are benign, while about 20% are malignant [5]. Malignant tumors grow rapidly and infiltrate surrounding brain tissue, whereas benign tumors spread more slowly. Benign tumors can also pose risks due to their expansion, which may impact adjacent brain tissue [6]. Thus, timely identification of brain tumors is crucial because of their life-threatening nature. According to the International Association of Cancer Registries, about 28,000 individuals in India are diagnosed with brain cancer annually, of whom over 24,000 die each year [7]. Other reports state that in the United Kingdom, 52,500 deaths have been recorded annually due to brain tumors [8]. In the United States, brain tumors impact a large portion of the population compared to many other countries. For example, in 2019, the total number of cases for both types of brain tumors was approximately 86,970 [9]. According to a study in 2021, it was estimated that in the United States, 24,530 adults were expected to be diagnosed with cancer-causing tumors in the brain and spinal cord. Radiologists use different procedures to diagnose brain tumors, which include biopsy, analysis of an X-ray, and analysis of cerebrospinal fluid. A biopsy is a surgical procedure in which a small piece of tissue is removed. Radiologists typically take two to four hours classifying the tumor, making it a time-consuming process [10]. Many factors are associated with biopsy, such as severe bleeding and inflammation. Furthermore, X-ray radiation within the skull may increase the risk of cancer [11]. Currently, radiologists are more interested in non-invasive imaging modalities due to their high diagnostic accuracy and minimal patient risk. Among these, MRI is considered the most effective for producing high-resolution images. Moreover, a CAD method is introduced that can recognize tumors in the brain at the primary stages without human involvement. This approach provides encouraging results in MRI-based diagnosis, supporting radiologists in making informed clinical judgments. ML and DL have significantly improved the computer-aided diagnosis (CAD) process in medical imaging [12,13]. Integrated machine learning techniques involve four key stages: preprocessing, segmentation, feature extraction, and classification. The preprocessing techniques include filtration, intensity correction, and skull stripping. Segmentation methods use thresholding, clustering, texture-based, and contour-based approaches to extract features and isolate the tumor region on the human skull [14]. These are basic methods used to identify features in MRI images, followed by a feature selection process to retain the most relevant features. However, feature extraction may sometimes lead to the loss of critical information from the original image. DL techniques have shown promising results in addressing this limitation. Using deep learning techniques, features are extracted automatically. CNNs consist of multiple layers designed to extract features from images [15]. Thus, CNNs are well-suited for large datasets [16]. However, obtaining large, annotated datasets can be quite challenging in medical image analysis. The convolution, pooling, and fully connected layers are part of the feed-forward architecture that makes up CNN structures. Feature extraction is performed using the first two types, and classification is carried out by the third. Examples of such architectures include AlexNet, VGGNet, MobileNet, DenseNet, Inception, ResNet, and NASNet [17]. A recent development in deep learning is transfer learning, which significantly improves performance in various pattern recognition tasks by leveraging features learned from previously trained CNN models [18,19]. Using this approach, several models have recently been developed based on popular architectures such as ResNet, Xception, and MobileNet [15]. In 2020 and 2021, brain tumor detection research using MRI datasets primarily focused on binary classification, distinguishing between normal brain scans and those with tumors. Preceding this period, prior research, as documented in several published papers [20], encompassed a broader scope. However, from 2021 onward, there has been a clear shift towards more comprehensive studies on segmentation and multi-class diagnosis of brain tumors. In 2025 and 2026, the trend appears to continue with increased emphasis on refining segmentation and advancing multi-class classification methods for brain tumor detection. Accurate detection in multi-classification tasks requires further investigation to achieve optimal performance. In this study, we propose two models for brain tumor multi-classification, a novel customized CNN and an optimized ResNet101 architecture. The key contributions of our work are as follows:

- Proposed a novel customized CNN with dynamic dropout and batch normalization, specifically designed to enhance tumor feature learning and reduce overfitting.
- Developed a strong training strategy with checkpointing and adaptive learning rate modifications to maximize convergence.
- Attained cutting-edge performance (97.72%) on multiple MRI datasets, surpassing existing approaches.
- Validated on a large and diverse dataset of 3,264 MRI images, demonstrating improved generalizability and enhancing real-world diagnostic reliability.
- Refined ResNet101 using Global Average Pooling (GAP) and progressive dropout (0.4 → 0.2) to improve feature retention and regularization in brain tumor identification.
- Incorporated batch normalization after GAP to stabilize gradient flow and enhance transfer learning efficiency.
- Enhanced high-level tumor biomarker representation by adding dense layers (128 → 64) with ReLU activation.
- Achieved 98.73% testing accuracy and 99.91% training accuracy, confirming superior model performance.
- Customized ReduceLROnPlateau (patience = 5 epochs) to prevent premature convergence and adapt to the complexity of MRI data, achieving a 95% F1-score on a 4-class dataset.

## Literature review

Over the past 20 years, different techniques based on DL and ML have been proposed for brain tumor diagnosis. An overview of some of these studies is provided in this section.

### Machine learning methods for brain tumor classification

Researchers are employing various ML techniques for brain tumor detection to extract features, reduce dimensions, and achieve accurate classification. Many authors proposed models for classifying benign and malignant tumors. Kharrat et al. [21] applied a support vector machine (SVM) combined with a genetic algorithm for binary brain image classification using spatial gray level dependency techniques for feature extraction. Another study by Bahadure et al. [12] segmented normal and abnormal brain tissues using Berkeley wavelet transformation and classified them using SVM, achieving 96.5% prediction accuracy on 135 images. Rehman et al. [11] have used an RF classifier on the BRATS dataset of 2012 and have given better precision and specificity than other classifiers. Chaplot et al. [22] the authors used Discrete Wavelet Transform (DWT) for feature extraction and SVM for classification. In the experimentation for the prediction of 52 images, they achieved accuracy up to 98%. El-Dahshan et al. used KNN as a classifier on 70 images; in prediction, the accuracy achieved was 98.6%. Anantharajan et al. [23] preprocessed brain tumor images using a median-based filtering technique combined with adaptive contrast enhancement, followed by fuzzy C-means segmentation. From the segmented regions, statistical features such as entropy, textural strength, brightness average, and variability were derived using a gray-level co-occurrence approach. In this study, the authors performed classification using SVM with a neural network, achieving an accuracy of 97.93%.

### CNN methods for brain tumor classification

Mohsen et al. [24] employed a DNN algorithm to categorize four categories of brain tumor images: sarcoma, metastasis, non-tumor, and glioblastoma. The authors used a dataset of 66 images, which was collected from Harvard. They obtained an accuracy of 96.97% with a DNN using extreme learning features. Deep learning methodology was given by Amjad Rehman et al. [25] to detect and characterize the microscopic brain tumors. Their approach was based on a handcrafted architecture of a 3D CNN to take out the tumor’s characteristics and makes use of a pre-trained CNN algorithm for the extraction of features. Using correlation-based selection, extracted features were then selected and added into a feed-forward neural network for categorization. On BraTS dataset, their model could thus reached accuracy of 98.32%, 96.97%, and 92.67% from 2015, 2017, and 2018, respectively. Goriparthi et al. [18] used a CNN architecture for a three-class brain tumor diagnosis system, achieving 98% training and 96% testing accuracies. Using CNN, Remak E. (2021) [15] developed a system for multiclassifying brain tumors to aid in the first diagnosis. Subba et al. [26] used CNN with the attention module to classify tumors into three types. Attention module was used to increase the performance of classification. The authors used Figshare MRI dataset with 3064 samples and achieved an accuracy of 97.62%. Gencer et al. [27] employed EfficientNetB0 in combination with a quantum genetic algorithm to create a hybrid model for the categorization of brain tumors, attaining an accuracy of 98.36%. Suryawanshi et al. [28] used VGG19, CNN and its hybrid model, combining CNN and SVM for brain tumor classification. In this study, authors achieved an highest accuracy of 96. 23% using the CNN-SVM hybrid model. Saeedi et al. [29] employed a 2D CNN and a modified convolutional autoencoder network for the classification of tumors. In this study, a dataset of 3,264 MRI images was used, which was then augmented three times for this purpose. The study obtained an accuracy of 93.44% using the 2D CNN and 90.92% with the convolutional autoencode. Dutta et al. [30] employed a CNN with an attention multiscale to perform multi classification of brain. The authors achieved 97.11% and 96.64% on BraTS 2020 and MBTD datasets. Kukreja et al. [31] used CNN with explainable AI to perform classification into three types. In this study, the authors used 6 convolutional layers in CNN and achieved an accuracy of 94.47%. Mzoughi et al. [32] employed CNN and vision transformer (VIT) to classify brain tumors using an MRI dataset. In this study, the authors achieved accuracies of 83.37% and 91.61% using the CNN and VIT models, respectively. Aamir et al. [33] used CNN to categorize brain tumors. For this purpose, the model was fine-tuned, complexity of this model was reduced, and the performance was enhanced. The model was trained using three publicly available kaggle datasets. In this study, average accuracy of 97% was achieved.

## Methodology

Brain tumors can be challenging to model due to similarities in the appearance of tumor and normal brain tissue. Additionally, tumors may vary significantly in size, shape, and intensity, making it harder to distinguish them from normal tissue in medical images. Accurate brain tumor diagnosis is crucial for efficient treatment planning and better patient outcomes. Deep learning, especially CNNs, has been successful in developing computer-assisted tumor detection systems. CNNs are a pivotal deep learning technique used extensively for image and video processing. Leveraging the principles of deep learning, CNNs perform both generative and descriptive tasks, making them indispensable in fields such as computer vision, recommender systems, and natural language processing (NLP).The human visual brain served as the inspiration for CNNs, are especially well-suited for applications that need grid-like data structures, such as images. Unlike traditional neural networks, CNNs use a specialized architecture to process data in a hierarchical manner, significantly reducing computational requirements and enhancing efficiency. Convolutional, pooling, and fully connected layers are the three primary parts of the CNN model. This section describes the brain tumor classification using two novel deep learning techniques. Initially, we utilized a novel customized CNN model tailored specifically for detecting brain tumors. we then applied an optimized ResNet101 model to the same dataset. ResNet101 is a well-known architecture renowned for its deep residual learning capabilities, which are advantageous for complex image recognition tasks. In the next sections, we provide a through examination of each component of our proposed models.

### Dataset

**Fig 1:**
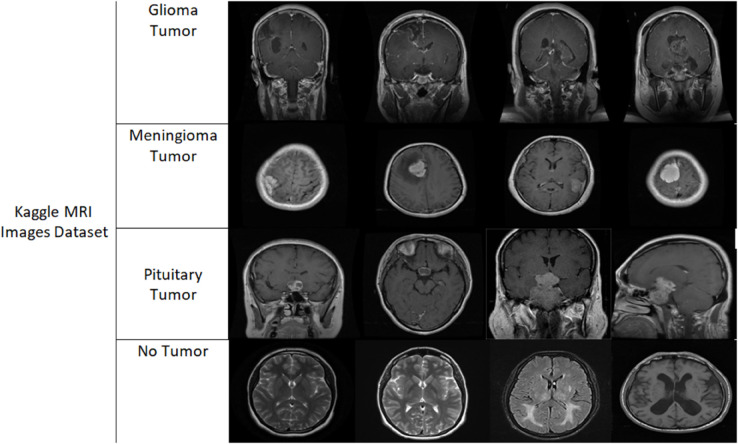
Samples of different brain tumor images.

In this work, we employed an open-access dataset sourced from Kaggle, created especially to find brain malignancies. The dataset contains 3,264 MRI slices, each representing brain scans from a diverse patient population. These MRI images are carefully classified into four classes: glioma tumors, meningioma tumors, pituitary tumors, and no-tumor cases [34]. The inclusion of multiple tumor types, alongside non-tumor examples, provides a comprehensive and diverse dataset, reflective of real-world clinical scenarios. This heterogeneity is vital for training deep learning models, as it facilitates a robust understanding of varying tumor characteristics and enhances the models’ ability to generalize across different cases. Consequently, this dataset is essential in both the development and evaluation phases of our proposed approach, ensuring its applicability to a broad range of brain tumor detection tasks. Fig 1 presents the sample of the brain images from various classes. Further details of the dataset, including the distribution of images across each category, are provided in Table 1.

**Table 1:** MRI image dataset distribution.

Dataset	Brain Tumor Categories	Training	Validation	Testing
Kaggle	Glioma Tumor	733	93	100
Kaggle	Meningioma Tumor	726	96	115
Kaggle	No Tumor	344	51	105
Kaggle	Pituitary Tumor	740	87	74

### Data preprocessing

We implemented several structured preprocessing steps to ensure optimal data quality and model performance. First, MRI images were read from designated directories using OpenCV’s cv2.imread() function. These images were then resized to a consistent dimension of 150x150 pixels with cv2.resize(), standardizing the input size for the Convolutional Neural Network (CNN) and ensuring uniformity across the dataset. Following image resizing, the images were labeled according to their directory names, which correspond to various tumor types or the absence of tumors. Labels include glioma tumor, pituitary tumor, meningioma tumor, and no tumor. This labeling is essential for supervised learning because it provides the essential ground truth to train the model. To avoid any potential biases introduced by the order of the images, we shuffled the dataset using the shuffle() function from sklearn.utils. Shuffling randomizes the order of the data, which helps prevent the model from learning patterns that might be artifacts of the original data sequence. The dataset was further divided into training and testing parts. Additionally, the model was trained and validated using 88% of the data, with the remaining 12% for testing. This division allows the model to be trained on a substantial portion of the data while evaluating its performance on unseen data. Then the labels were converted from categorical string values to numerical indices. Because it transforms the labels into a binary matrix format, this transformation is crucial for the multi-class classification’s categorical cross-entropy loss function.

### A novel customized CNN architecture

**Fig 2:**
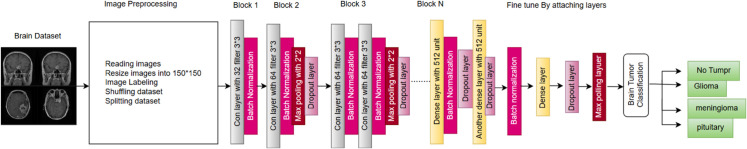
Proposed novel customized CNN architecture for brain tumor classification.

In this study, we first used a CNN model and fine-tuned its parameters to categorize brain MRI images into four classes: meningioma tumor, pituitary tumor, glioma tumor, and non-tumor. We refer to this model as a novel customized CNN model. The architecture of the novel customized CNN was carefully adjusted to enhance its performance. Fig 2 presents the architecture of the novel customized CNN employed for brain tumor detection. One of the core components of a CNN model is the convolutional layer. Convolution is the process of applying a kernel or filter to an input array to produce a transformed feature map, capturing important patterns or features. In our study, we used 9 convolutional layers with the same kernel size of 3×3. This specific depth was chosen to enable hierarchical feature extraction, allowing the model to capture both low-level and high-level spatial features critical for accurate brain tumor classification. Using the same kernel size across all convolutional layers ensures consistent feature extraction and helps the network learn patterns uniformly. The activation function employed in this study was ReLU, which helps to solve the vanishing gradient problem. The definition of the ReLU is as follows:

f(z)=max(0,z)

Additionally, 4 max pooling layers were employed in our model in order to decrease the feature maps’s spatial dimensions, thereby minimizing computational complexity and enhancing the network’s ability to generalize. Batch normalization is also an essential component to design the CNN model. In this study we utilized 14 batch normalization layers throughout the network to ensure optimal training dynamics and model accuracy. Batch normalization mitigates overfitting by incorporating a regularization effect and enhancing gradient flow. Furthermore, we used six dense layers along with the output layer’s softmax activation function for classification. To solve the issue of overfitting in deep learning models, we incorporated dropout layers before the softmax function in our novel customized CNN architecture.

### Optimized Resnet101 architecture

**Fig 3:**
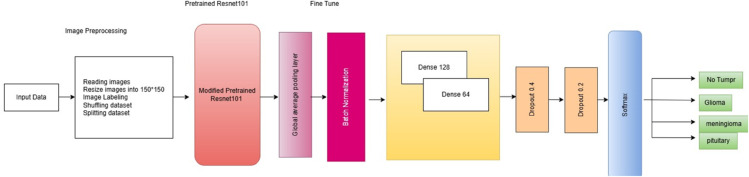
Proposed optimized ResNet101 architecture for brain tumor classification.

In our study, we utilized a pre-trained ResNet101 model as a second approach, which was fine-tuned to classify the tumor into four classes. Fig 3 presents the architecture of optimized ResNet101 for brain tumor detection. We modified the ResNet101 model during training for our specific task and refer to it as optimized ResNet101. We first removed the top fully connected layers, retaining only the convolutional base. This allowed us to use ResNet101 as a powerful feature extractor. Next we applied a Global Average Pooling (GAP) layer to reduce the spatial dimensions of the feature maps, effectively converting them into a 1D vector for each feature map. Gap was chosen to reduce the number of trainable parameters, thereby minimizing the risk of overfitting while preserving spatial features critical for brain tumor classification. The Batch Normalization layer was integrated after the GAP to enhance the speed and stability of the training. Additionally, two Dropout layers with rates of 0.4 and 0.2, respectively, were integrated in a progressive manner, placing the higher rate earlier to regularize the model and minimize the risk of overfitting. To further increase the model’s functionality to learn complex representations, two dense layers were added, one with 128 units and the other with 64 units. Both layers used the ReLU activation function. These layers allow the model to better identify the patterns in the data. Additionally, a dense layer with four units and a softmax activation function makes up the final output layer. The Adam optimizer was utilized to compile the entire model, chosen for its efficiency in training deep learning models.

## Results and discussion

We modified both the CNN and ResNet architectures, fine-tuned these models on the same dataset by adding extra layers and adjusting various parameters, and evaluated their performance in classifying tumors into four categories. The performance was evaluated using various performance metrics. The following section provides the details of the experimental setup, performance metrics, results, and discussion.

### Training and hyperparameters of novel customized CNN

The parameters chosen for training the modified CNN model are presented in Table 2. There are various optimizers used to train deep learning models; among them, the Adam optimizer was used with the default learning rate. To dynamically adjust the learning rate during training, the ReduceLROnPlateau callback was employed. This callback monitors validation accuracy and, after a predetermined number of epochs, decreases the learning rate by a factor of 0.1 if no improvement is seen. The model was trained for 35 epochs, as illustrated in Fig 2. To further evaluate the generalization performance of the novel customized CNN, a 5-fold cross-validation was employed to the 88% of the dataset allocated for training and validation. Table 3 presents the fold-wise training and validation accuracies. The model achieved an average training accuracy of 99.03 ± 0.01% and an average validation accuracy of 96.31 ± 0.01 across the five folds. The best-performing fold with the highest training and validation accuracy was then selected for final evaluation on an independent test dataset. Using the best model, the proposed customized CNN achieved training and testing accuracies of 99.05% and 97.72%, respectively.

**Table 2:** Hyperparameter settings of the novel customized CNN.

Hyperparameter	Setting
Loss Function	Categorical Crossentropy
Epochs	35
Optimizer	Adam
Metric	Accuracy
Learning Rate	Default (0.001)
Batch Size	36

**Table 3:** Fold-wise training and validation accuracies of the novel customized CNN.

Fold	Training Accuracy (%)	Validation Accuracy (%)
1	99.03	96.30
2	99.02	96.31
3 (Best Fold)	99.05	96.33
4	99.04	96.32
5	99.03	96.30
**Average ± Std**	**99.03 ± 0.01**	**96.31 ± 0.01**

### Training and hyperparameters of optimized Resnet101

The optimized ResNet101 model was trained on 35 epochs using the 64 batch size. Similarly to the novel customized CNN, the Adam optimizer with default values is used in the optimized ResNet101. The hyperparameter setup of optimized ResNet101 is shown in Table 4. Similarly, 5-fold cross-validation was also performed on 88% of the data allocated for validation and training to assess the generalization performance of optimized ResNet101. Table 5 presents the fold-wise training and validation accuracies. The model achieved an average training accuracy of 99.87 ± 0.03% and an average validation accuracy of 97.23 ± 0.03%. The small standard deviation in both metrics indicates the model’s robustness and stability, which suggests no sign of overfitting or underfitting in any individual fold. For the final evaluation the best fold (the fold with the most accurate validation accuracy) was selected and evaluated using a 12% test set. Using the best model, the optimized ResNet101 achieved training and testing accuracies of 99.91% and 98.73%, respectively.

**Table 4:** Hyperparameter settings of the optimized ResNet101.

Hyperparameter	Setting
Loss Function	Categorical Crossentropy
Epochs	35
Optimizer	Adam
Metric	Accuracy
Learning Rate	Default (0.001)
Batch Size	64

**Table 5:** Fold-wise training and validation accuracies of the optimized ResNet101.

Fold	Training Accuracy (%)	Validation Accuracy (%)
1	99.83	97.18
2	99.85	97.23
3 (Best Fold)	99.91	97.28
4	99.86	97.21
5	99.89	97.24
**Average ± Std**	**99.87 ± 0.03**	**97.23 ± 0.03**

### Performance matrices

To evaluate the performance of our best-performing optimized ResNet101 and compare it with previous studies, we utilized various metrics. These matrices provide a thorough understanding of how well each model classifies brain tumors. The definitions of the metrics used in our evaluation are as follows:

Accuracy: Out of all the samples, it calculates the percentage of accurately classified samples (including TP and TN). It gives a general indication of the model’s performance in every class.

$$\text{Accuracy}=\frac{TP+TN}{TP+TN+FP+FN}$$ (1)

Recall: Recall is the ratio of true positive predictions to the sum of true positives and false negatives.

$$\text{Recall}=\frac{TP}{TP+FN}$$ (2)

Precision: It is determined by dividing the total number of positive predictions, including both false positives and genuine positives, by the percentage of true positive predictions. The following is the formula:

$$\text{Precision}=\frac{TP}{TP+FP}$$ (3)

F1 Score: The F1 score is a statistic that calculates the harmonic mean of precision and recall to combine them into a single value. It is particularly useful when evaluating models on datasets that are unbalanced. The F1-score calculation involves:

$$F1=2\times \frac{\text{Precision}\times \text{Recall}}{\text{Precision}+\text{Recall}}$$ (4)

### Results

**Fig 4:**
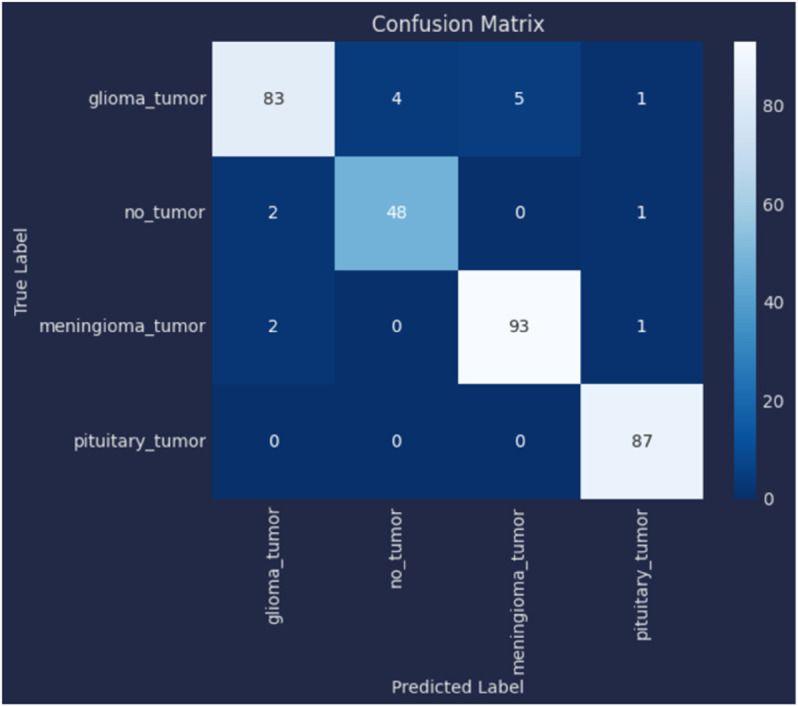
Validation confusion matrix of the optimized ResNet101 model.

**Fig 5:**
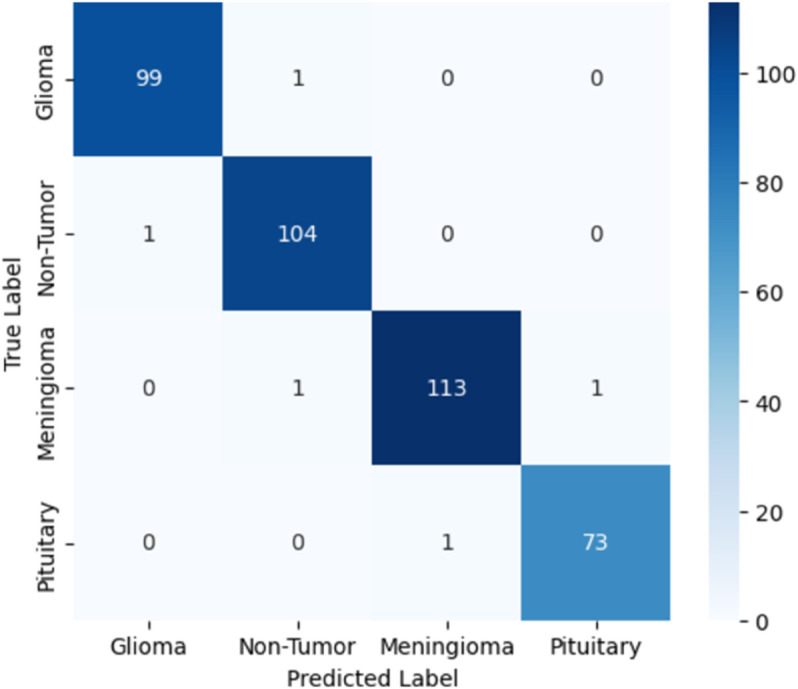
Testing confusion matrix of the optimized ResNet101 model.

**Fig 6:**
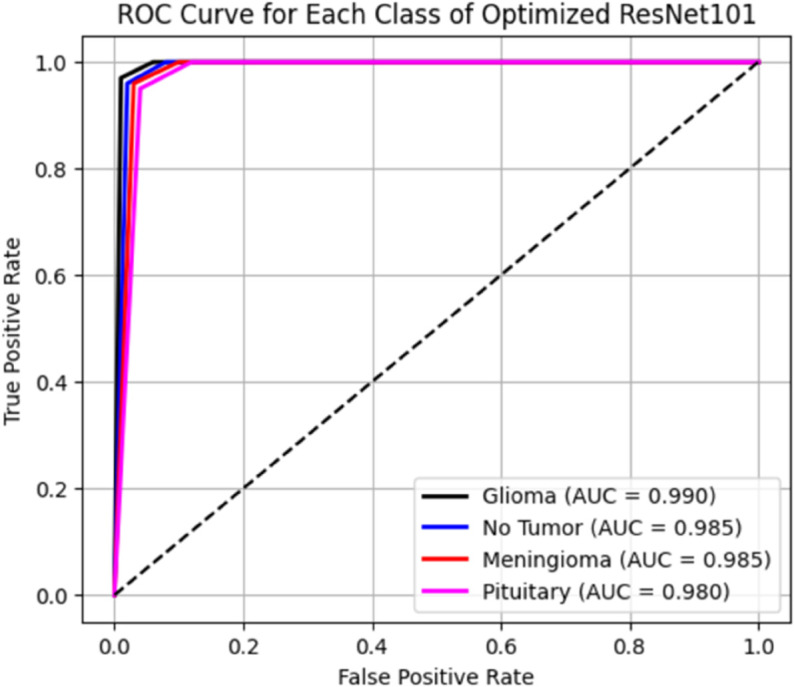
Roc curve illustrating the AUC for the optimized ResNet101.

**Fig 7:**
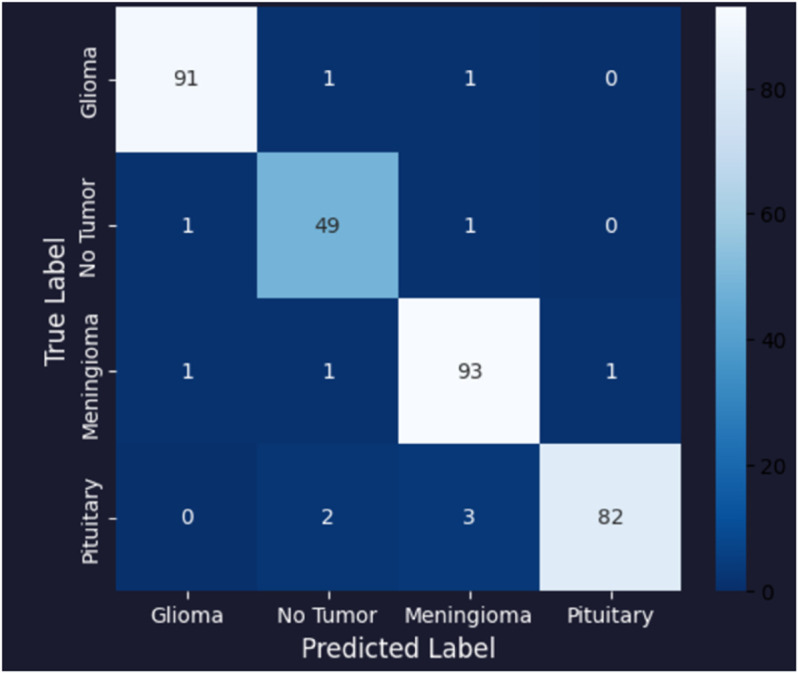
Validation confusion matrix of the novel customized CNN.

**Fig 8:**
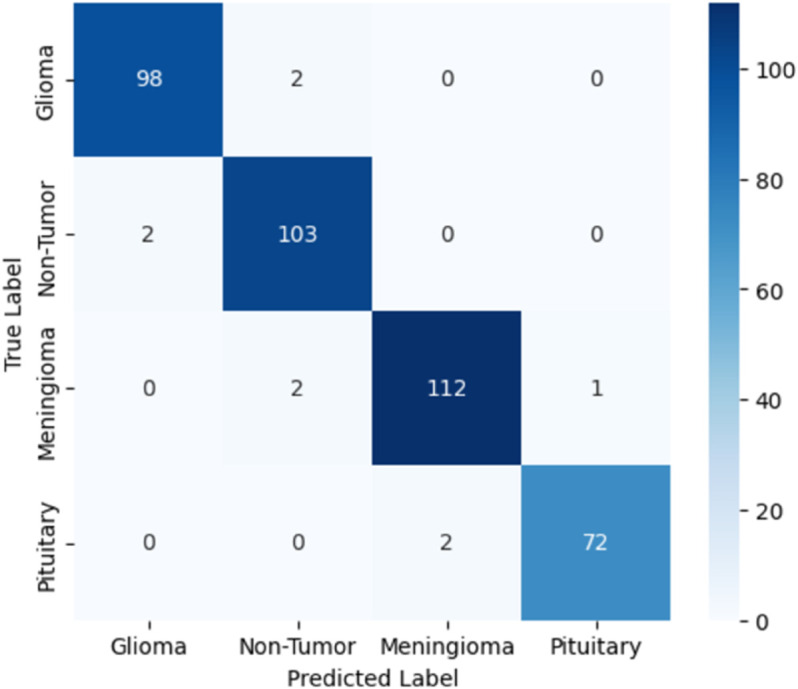
Testing confusion matrix of the novel customized CNN.

**Fig 9:**
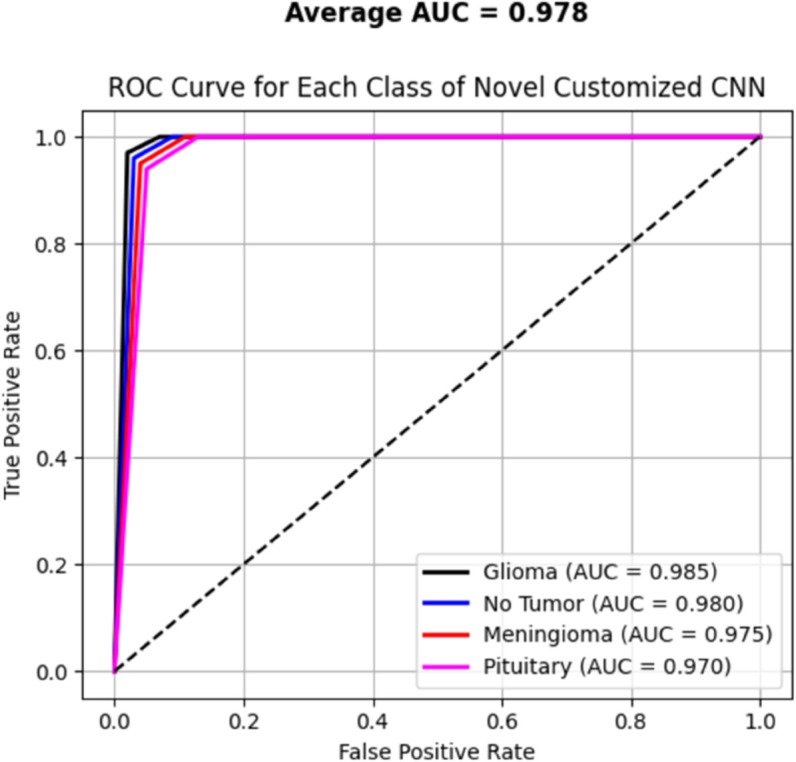
Roc curve illustrating the AUC for the novel customized CNN.

Table 6 presents the results of our proposed models, specifically comparing a novel customized CNN and optimized ResNet101 models. We initially applied a novel customized CNN model, fine-tuning its parameters to classify brain tumor data into four classes. Subsequently, we employed an optimized ResNet101 model, also fine-tuned on the same dataset for the same classification task. To ensure generalization performance, both these models were evaluated using 5-fold cross validation. The best-performing fold was selected based on validation accuracy, and all results, including training and validation loss curves, accuracy curves, confusion matrix, ROC curves, and AUC, are reported from the best fold. Our experiments showed that the optimized ResNet101 achieved superior performance in terms of accuracy and all reported metrics as compared to the novel customized CNN. The comparison of both these models with other studies is illustrated in the discussion section. The validation and testing confusion matrix of the optimized ResNet101, which demonstrated superior performance, are shown in Fig 4 and Fig 5, respectively. Fig 6 displays the Area Under the Curve (AUC) values for each class as well as the Receiver Operating Characteristic (ROC) curves, confirming the model’s resilience. Further, the confusion matrix of our novel customized CNN is shown in Fig 7 and Fig 8, while Fig 9 shows the ROC curves with the AUC of each class.

**Table 6:** Performance comparison between the novel customized CNN and optimized ResNet101.

Model	Training Accuracy	Testing Accuracy
Novel customized CNN	99.05%	97.72%
Optimized ResNet101	99.91%	98.73%

In Fig 4, the validation confusion matrix of optimized ResNet101 is present. The model performed well on the validation set, achieving an F1-score of 0.95, along with high precision and recall for each class. Notably, the model’s dependability was demonstrated by Pituitary Tumor (Class 3) achieving a flawless recall of 1.00. Fig 5 presents the testing confusion matrix of the optimized ResNet101 model, which shows how well it classified the four tumor classifications. The model demonstrated strong precision and recall for glioma tumors, accurately classifying 99 samples and misclassifying only one as no tumor. One sample was incorrectly labeled as glioma, whereas 104 samples were correctly classified, indicating that no tumor cases were also well-identified. The model performed well in identifying meningioma tumors, correctly identifying 113 cases, while incorrectly identifying one as a pituitary tumor and one as no tumor. Finally, just one case of pituitary tumor was misclassified as meningioma tumor, out of 73 accurate predictions. Overall, the model achieved a balanced performance with precision, recall, and F1-score all at 0.99, reflecting its consistent effectiveness across different tumor types. These findings show that there are few misclassifications and that the model successfully differentiates between various tumor types. The resilience and great generalization of multi-class tumor classification are indicated by the good classification performance across all classes. With AUC values of 0.990 for gliomas, 0.985 for non-tumors, 0.985 for meningiomas, and 0.980 for pituitaries, with an average AUC of 0.985, Fig 6 further demonstrates the optimized ResNet101 model’s exceptional discriminative ability across all tumor types. In Fig 7, the validation confusion matrix of the novel customized CNN is presented. The model also demonstrated strong performance on the validation set. Lastly, Fig 8 presents the testing confusion matrix of the novel customized CNN model. The model also achieved strong precision and recall for glioma tumors, accurately classifying 98 samples and misclassifying only two as no tumor. Two samples were incorrectly labeled as glioma, whereas 103 samples were correctly classified, indicating that no tumor cases were also well-identified. The model performed well in identifying meningioma tumors, properly identifying 112 cases but incorrectly identifying one as a pituitary tumor and two as no tumor. Finally, two cases of pituitary tumor were misclassified as meningioma tumors, out of 72 accurate predictions. Overall, the model achieved a balanced performance with precision, recall, and F1-score all at 0.98. AUCs of 0.985 (glioma), 0.980 (non-tumor), 0.975 (meningioma), and 0.970 (pituitary) were achieved by the novel customized CNN, as shown in Fig 9. The pituitary class’s slightly lower result was due to its smaller sample size (n=74). The clinical reliability of both models were demonstrated by their strong alignment with their F1 scores (≥0.98) and testing accuracies (optimized ResNet101: 98.73%; novel customized CNN: 97.72%).

**Fig 10:**
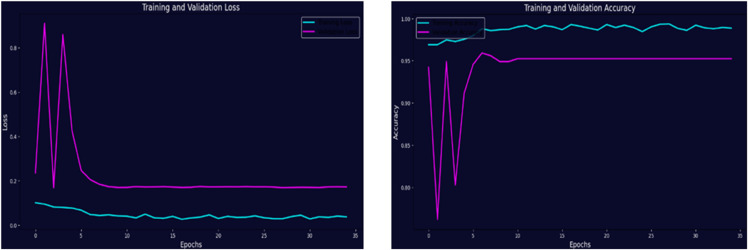
Training progress of the novel customized CNN.

**Fig 11:**
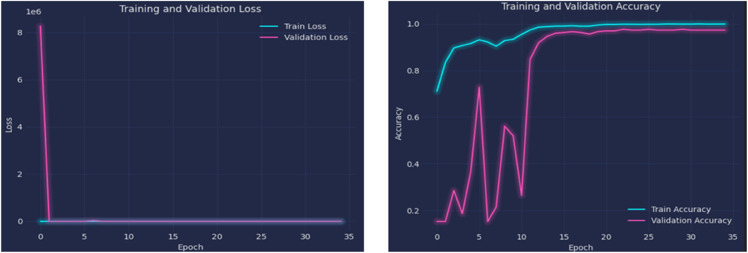
Training progress of the optimized ResNet101.

The left plot in Fig 10 represents the training and validation loss over 35 epochs. Initially, both training and validation losses were high during the early epochs. As the training progress was increased, the model learned effectively without overfitting. Furthermore, the right plot in Fig 10 presents the training and validation accuracy. The training accuracy improved and reached 99.05%, while the validation accuracy reached 96.33%. This demonstrates that the model performed well on unseen data. Further, the left plot in Fig 11 represents the training and validation loss of the optimized ResNet101. Initially, both losses were extremely high during the early epochs, but they rapidly declined and stabilized at near-zero values. This significant drop indicates that the model learned quickly and effectively minimized errors. The right plot illustrates the training and validation accuracy. The training accuracy improved steadily, reaching 99.91%, while the validation accuracy also performed well, achieving 97.28%. This demonstrates that the model generalizes effectively and performs well on unseen data. The improved generalizability of the optimized ResNet101, especially for minority classes, was probably influenced by its deep architecture and optimization. The optimized ResNet101 exhibited minor advantages in managing class imbalances and diagnostic edge cases, yet these results confirm both models as reliable models for classifying brain tumors. To summarize the key evaluation metrics of both these models, Table 7 compares their testing performance of both models. This summary highlights the advantage of optimized ResNet101 across all metrics.

**Table 7:** Testing results comparison between the novel customized CNN and the optimized ResNet101.

Model	Accuracy	Precision	Recall	F1-score	AUC
Novel customized CNN	97.72%	98%	98%	98%	0.978
Optimized ResNet101	98.73%	99%	99%	99%	0.985

### Discussion

In our study, we proposed two models for brain tumor multi-classification: a novel customized CNN and an optimized ResNet101. Both models were evaluated using the same dataset, and the results showed that optimized ResNet101 outperformed the novel customized CNN. The accuracy of the novel customized CNN increased from 86% to 97.72% as a result of training enhancement and key architectural improvement. Hierarchical feature extraction is made possible by a 9-layer deep convolutional stack with gradually increasing filters from 32 to 256, which captures fine-grained tumor patterns. Batch normalization reduces initial sensitivity and speeds up convergence by stabilizing gradient flow. Dynamic dropout 0.3 reduces overfitting and enhances noise robustness. ModelCheckpoint maintains the optimal model weights, while ReduceLROnPlateau prevents stagnation by adjusting learning rates. Finally, by creating discriminative embeddings, dense layers (512 → 64 units) adjust decision bounds. By improving generalization and addressing dataset bias and tumor heterogeneity, these advancements make CNN a useful tool for therapeutic settings. Further, The refined ResNet101’s impressive 98.73% testing accuracy is due to strong architectural improvements. In order to preserve spatial tumor features and minimize overfitting, Global Average Pooling (GAP) replaced the position of conventional flattening. Progressive dropout (0.4–0.2) successfully improved tumor-specific biomarkers and removed superfluous ImageNet features. In order to mitigate the "vanishing features" challenge, dense layers (128 → 64) fused high-level tumor characteristics, whereas batch normalization following GAP stabilized training against MRI intensity changes. By avoiding premature convergence and fine-tuning weight updates, the customized ReduceLROnPlateau (patience = 5, factor = 0.1) improved accuracy over the baseline ResNet101. The performance of both the novel customized CNN and the optimized ResNet101 was compared with existing studies, as detailed in Table 8. Both of our models demonstrated strong performance in comparison to previously reported methods, with the optimized ResNet101 achieving the highest accuracy and stability across all reported metrics. This comparison shows the superior performance of our optimized ResNet101 model and its valuable contribution to the medical imaging field.

**Table 8:** Performance comparison of the novel customized CNN and the optimized ResNet101 with existing studies.

Model	Accuracy	Precision	Recall	F1-score
CNN [35]	97.00%	95.00%	95.00%	95.00%
2D CNN [29]	93.44%	94.75%	95.75%	95.00%
ResNet50 [36]	87.21%	87.27%	87.14%	85.52%
Fusion model [36]	95.05%	96.13%	89.77%	94.95%
LeaSE+DARTS [37]	90.61%	91.49%	91.50%	91.48%
COA-CNN [38]	95.18%	89.44%	90.75%	90.61%
Transfer Learning [39]	97.06%	98.05%	97.02%	97.09%
M-CNN [40]	96.03%	96.00%	92.02%	96.02%
MobileNetV2 [41]	96.05%	98.02%	96.03%	97.04%
Our Customized CNN	97.72%	98%	98%	98%
Our Optimized ResNet101	98.73%	99%	99%	99%

## Conclusion

This study employed two deep learning models, a novel customized CNN and an optimized ResNet101 model, for accurate classification of brain tumors into four classes. Both of these models were employed to the same dataset, which was obtained from Kaggle. Our study demonstrates that both models perform effectively on this dataset, significantly enhancing the accuracy of brain tumor classification. Among both of these models, the optimized ResNet101 model outperformed the customized CNN across all evaluation metrics, achieving superior performance. We applied five fold cross-validation to ensure the robustness of these models. The novel customized CNN and the optimized ResNet101 achieved average training accuracies of 99.03±0.01% and 99.87±0.03% and average validation accuracies of 96.31±0.01% and 97.23±0.03%, respectively. Additionally, the best-performing fold was then selected and evaluated on the test set, achieving training accuracies of 99.05% and 99.91% and testing accuracies of 97.72% and 98.73% from the novel customized CNN and an optimized ResNet101 model, respectively. Therefore, we believe that both of these models demonstrate outstanding performance in brain tumor classification and contribute significantly to the field of medical imaging. Overall, the proposed models demonstrate strong potential for accurately classifying brain tumor types and provide valuable support for early diagnosis and clinical decision-making.

## Limitations and future work

While the proposed models show strong classification performance, this study has certain limitations. First, the dataset used in our study is limited in size and lacks demographic diversity, potentially limiting the applicability of the findings across various imaging conditions and patient populations. Second, external validation using independent or multi-center datasets is not performed. Additionally, class imbalance also has a minor impact on model performance for tumor classes, which are underrepresented. In future research, our aim is to evaluate these models on multi-center brain tumor datasets acquired from different hospitals in real clinical settings to further validate their robustness across diverse imaging sources. Clinical settings involve various imaging modalities, devices, and patient demographic variations that are not fully represented in our current dataset. So, we anticipate that our model’s performance may differ slightly in such an environment. We also aim to use federated learning to facilitate cooperative and secure model training across hospitals. Furthermore, we intend to integrate explainable AI to enhance the interpretability and transparency of the model. Integration of explainable AI will not only foster trust but also support confident decision-making in real-world settings.
